# Dr. J. Metcalf Polk

**Published:** 1904-05

**Authors:** 


Dr. J. Metcalf Polk, of New York, son of Dr. W. M. Polk, dean of the Cornell University Medical College, died March 29, 1904, at the home of his father. He was taken ill with a cold about ten days previously, and pneumonia developed, after which he was attacked with edema of the lungs, which caused his death. Dr. Polk had been practising only a short time, and was a member of the visiting staff of Bellevue Hospital.

